# Seroepidemiological investigation of HAdV-4 infection among healthy adults in China and in Sierra Leone, West Africa

**DOI:** 10.1038/s41426-018-0206-y

**Published:** 2018-12-05

**Authors:** Busen Wang, Jianhua Li, Shipo Wu, Yi Chen, Zhe Zhang, Yanfang Zhai, Qiang Guo, Jinlong Zhang, Xiaohong Song, Zhenghao Zhao, Lihua Hou, Wei Chen

**Affiliations:** 1 Beijing Institute of Biotechnology, 20 East Street, Beijing, 100071 China; 2 Key Laboratory of Cell Proliferation and Differentiation of the Ministry of Education, School of Life Sciences, Peking University, 5 Yiheyuan Road, Beijing, 100871 China

## Abstract

An apparent increase in the frequency of human adenovirus type 4 (HAdV-4) infections among general populations has been observed over the past 10 years. However, available epidemiological data that may reflect previous viral circulation and assist in predicting potential outbreaks are sparse, particularly in mainland China and Africa. In this study, a convenient neutralization assay for use in the surveillance of historical HAdV-4 infections was established based on a recombinant luciferase-expressing virus. Subsequently, the neutralizing antibodies (nAbs) of 1013 healthy adult serum samples from China and Sierra Leone were evaluated. Our results showed that over 50% of the participants from China and nearly 70% of donors from Sierra Leone had detectable nAbs against HAdV-4 despite the few infection cases officially reported in these regions. Furthermore, the prevalence of nAbs to HAdV-4 is lower than that to HAdV-5, and both varied by geographic location. In addition, the seropositive rates of both HAdV-4 and HAdV-5 nAbs increased with age. However, the nAbs stimulated by HAdV-4 remained stable at low (≤200) levels among the different age groups, whereas moderate (201–1000) or high (>1000) nAb levels were produced by HAdV-5 and tended to decrease with age. These results elucidate the human humoral immune response against HAdV-4 and revealed that this virus may be an underestimated causative agent of respiratory disease among adults in China and West Africa, demonstrating the importance of HAdV-4 surveillance and providing useful insights for the future development of HAdV-4-based vaccines.

## Introduction

Human adenoviruses (HAdVs) are nonenveloped, double-stranded DNA viruses belonging to the Adenoviridae family. To date, a total of 90 HAdV subtypes have been documented and classified into 7 species (A–G) according to their serological and molecular characteristics^1–3^. A variety of severe diseases with a broad range of symptoms, such as acute febrile respiratory disease, conjunctivitis, cystitis, gastroenteritis, myocarditis, and even life-threatening pneumonia are caused by HAdV infections in healthy adults and particularly in immunocompromised infants or young children^2,4^.

HAdV species B (HAdV-B3, B7, B21, and B55) and C (HAdV-C1, C2, C5, and C6) are the most common viruses responsible for severe respiratory infections worldwide^1,5–11^. HAdV-4, the only member of human adenovirus species E, has been a significant cause of acute respiratory disease in the US military recruits since its identification in the 1950s^12^. The high susceptibility to HAdV-4 infection and the high communicability among incoming recruits during basic training might be attributable to the low level of pre-existing immunity in these individuals as well as the closed and crowded conditions in barracks^12^. However, outbreaks of severe acute febrile respiratory disease and even pneumonia due to HAdV-4 infections have recently occurred among populations of children in India, Taiwan, and South Korea^1,2,5,13,14^, as well as in healthy immunocompetent adults in the United States, Italy, and Singapore^15–17^. Increasing attention has been paid to HAdV-4-associated illnesses during the past 6 decades. However, although limited epidemiological data exists in the United States, no similar reports are available elsewhere, especially in China and Africa.

In this study, we established a specific HAdV-4 neutralization assay based on a recombinant replication-competent HAdV-4-luciferase (Ad4-Luc) virus to study the distribution of nAbs in relation to sex and age. In addition, we assessed the cross-reactivity and analyzed the differences in nAb levels between HAdV-4 and HAdV-5, the most representative serotype of prevalent adenovirus species C, which will greatly increase our understanding of the circulation of HAdV-4 and of human immunity to this pathogen.

## Materials and methods

### Generation of recombinant HAdV-4 and HAdV-5 reporter viruses

The recombinant HAdV-5 vector-based luciferase-expressing virus was constructed using the AdMax system (Microbix Biosystems, ON, Canada). Human adenovirus type 4 strain RI-67 (GenBank No. AY594253) was purchased from the American Type Culture Collection (Manassas, VA, USA), and recombinant replication-competent adenovirus HAdV-4 expressing luciferase protein was constructed according to published protocols^18^. Briefly, the terminal regions of the HAdV-4 genomes were polymerase chain reaction (PCR) amplified and subcloned into the pET-28a vector (conferring kanamycin resistance; Novagen, WI, USA) to obtain shuttle plasmids. The genomic plasmid pAd4 was generated by homologous recombination between the HAdV-4 genome and the shuttle plasmids in *Escherichia* *coli* BJ5183 competent cells (Agilent Technologies, CA, USA).

The open reading frame of the ampicillin resistance gene and the fragments flanking the E3 region were then PCR amplified and subcloned into pET-28a to obtain pAd4-E3L-Amp-E3R. After sequencing, the E3L-Amp-E3R fragment was excised from pAd4-E3L-Amp-E3R and transformed into BJ5183 competent cells containing pAd4. The E3 region in pAd4 (conferring kanamycin resistance) was then deleted by homologous recombination with the E3L-Amp-E3R fragment using ampicillin screening to obtain pAd4ΔE3-Amp (conferring kanamycin and ampicillin resistance). The ampicillin fragment was subsequently deleted using the restriction enzyme *Spe*I (New England Biolabs, MA, USA) to obtain pAd4ΔE3. The luciferase expression cassette with the CMV promoter and the SV40 poly-A tail was PCR amplified and inserted into pAd4ΔE3 to obtain pAd4ΔE3-Luc using a Gibson Assembly Cloning Kit (New England Biolabs, MA, USA).

The recombinant Ad4-Luc virus was rescued by transfection of the linearized pAd4ΔE3-Luc plasmid into HEK293 cells and was propagated in A549 cells. Positive recombinant adenovirus was propagated in HEK293 cells and purified by ion-exchange chromatography on a Source 30Q column (GE Healthcare, Beijing, China). The HAdV-4 viral titers were determined in HEK293 cells using the same method as the Adeno-X^TM^ Rapid Titer Kit (Clontech, CA, USA), with the exception that the primary and secondary antibodies used were mouse anti-adenovirus Hexon (AbD Serotec, Kidlington, UK) and Goat Anti-Mouse IgG2a (Abcam, Cambridge, UK). Briefly, 50 μL of 10-fold serial dilutions of viral samples (from 10^−3^ to 10^−5^) were added to 0.5 mL of HEK293 cells (5 × 10^4^cells/mL) in each well of 24-well plate. After incubating at 37 °C for 48 h, the supernatants were discarded and 0.5 mL of ice-cold 100% methanol was added to each well to fix the cells for approximately 10 min at −20 °C. The wells were gently rinsed three times, after which the primary antibody was added and incubated at 37 °C for 1 h. Next, the wells were rinsed three times and the secondary antibody was added and incubated at 37 °C for 1 h. Finally, the DAB working solution (Abcam, Cambridge, UK) was used to visualize the infected cells. Positive cells were counted using a microscope, and infectious units (IFU)/mL for each sample was calculated as (infected cells/field) × (field/well)/(volume virus (mL) × (dilution factor)).

### Human serum collection

Serum samples were collected from 1013 healthy adults living in China and Sierra Leone. Of these samples, 255 samples were obtained in March 2017 from Beijing, the capital city of China; 260 samples were obtained throughout the year 2015 from Jiangsu, the eastern coastal province of China; and 498 samples were obtained in October 2015 from Freetown, the capital city of Sierra Leone in West Africa. The ages of the participants ranged from 18 to 65 years old. The collection and use of the serum samples for this study was approved by our institutional review boards, and informed consent was obtained from each participant. Samples collected in Sierra Leone were involved in the clinical trial of an HAdV-5-based vaccine. The study was approved by the Sierra Leone Ethics and Scientific Review committee as well as the Pharmacy Board of Sierra Leone and was performed in accordance with the Declaration of Helsinki.

### Adenovirus neutralization assays

**Fig. 1:**
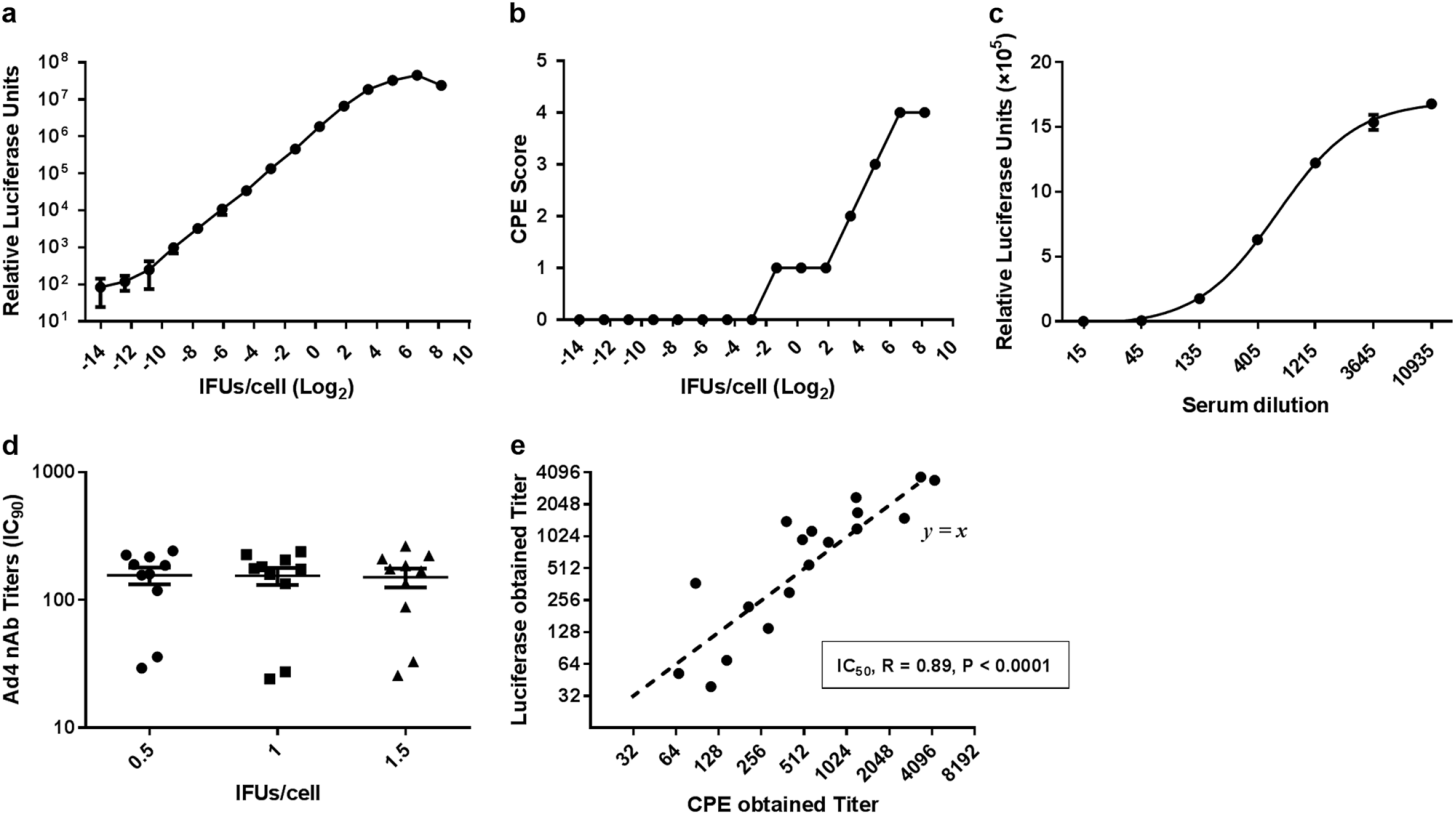
The establishment and validation of neutralization assays based on an Ad4-Luc recombinant virus. Serial dilutions of the Ad4-Luc recombinant virus were added to 2×10^4^ A549 cells in a 96-well plate (**a** and **b). a** The CPE score was divided into 5 levels (0–4) that represent approximately 0, 25, 50, 75, and 100% of cells exhibiting cytopathic effects after 1 day of viral infection, respectively. **b** Luciferase expression was also measured at 24 h postinfection. **c** Serial dilutions of HAdV-4-positive serum samples were incubated with Ad4-Luc and then were used to infect A549 cells. The luciferase assay was performed at 24 h postinfection. **d** Ten HAdV-4-positive serum samples were incubated with Ad4-Luc at three fixed IFU/cells ratios (0.5, 1, and 1.5). After the neutralization assay was performed, serum titers were calculated by the dilution level at which 90% of luciferase expression was inhibited. The difference between the three groups was analyzed by a one-way ANOVA test. **e** HAdV-4-positive serum samples were tested by two different neutralization assays, and the IC_50_ values obtained by the luciferase expression inhibition assay (*y*-axis) and the cytopathic effect assay (*x*-axis) were compared and analyzed for Spearman’s correlation coefficient

HAdV-5-specific nAbs were assessed by a luciferase-based virus neutralization assay as described previously^19^. In brief, serum samples were heat-inactivated at 56 °C for 60 min before a serial triple dilution was performed in 96-well tissue culture plates. The final serum dilutions were 1:12, 1:36, 1:108, 1:324, 1:972, 1:2916, and 1:8748, and each serum sample was tested in duplicate, and diluent without serum was used as a negative control. Subsequently, 50 μL of each serum dilution was mixed and incubated with 50 μL of Ad5-Luc at 1 × 10^7^ viral particles per well for 1 h at 37 °C. Next, 100 μL of 2 × 10^4^ A549 cells was added into the mixture and incubated for 24 h at 37 °C, after which the supernatant was discarded and luciferase expression was detected using the Luciferase Assay System (Promega, Madison, WI). Similarly, HAdV-4-specific nAbs were tested using the Ad4-Luc recombinant virus described above, except that the viral load was set at 2 × 10^4^ IFU per well and final serum dilutions of 1:4 to 1:2916 were used. The final Ad4-Luc viral load was determined by changes in the cytopathic effects and luciferase expression levels, which corresponded to the amount of virus (Fig. 1). For both the HAdV-4 and HAdV-5 viruses, the nAb titer was calculated as the reciprocal of the dilution for which luciferase activity was reduced by 90% of that of the negative control (IC_90_) using nonlinear regression with four parameters in GraphPad. Values <12, 12–200, 201–1000, and >1000 were defined as negative, low, moderate, and high nAb titers, respectively.

To verify the accuracy of the luciferase-based HAdV-4 neutralization assay, a traditional cytopathic effect assay was also used to determine HAdV-4 nAb titers. In brief, the same serial dilutions of the inactivated serum samples were mixed with Ad4-Luc (100 TCID_50_ per well) and incubated for 1 h at 37 °C. Subsequently, 2.5 × 10^4^ HEK293 cells per well were added to the 96-well plate, and after 6 days of incubation, cell viability was assessed with a Cell Proliferation Assay kit (Promega, Madison, WI). HEK293 cells were used in the cytopathic effect assay due to their greater susceptibility to HAdV-4, resulting in pronounced cytopathic effects.

### Data analysis

The neutralizing antibody titers measured under different conditions were compared using a one-way ANOVA test. The Spearman correlation coefficient was calculated to analyze the correlation between the luciferase activity- and cell viability-based neutralization methods. Seroprevalences in different groups were compared using the Pearson chi-square test. Comparisons of nAb titers among groups were performed by the Mann–Whitney test. The trends in adenovirus seropositive rate versus age were analyzed by Cochran–Armitage trend tests, and the trends in adenovirus nAb titers among age groups were analyzed by Jonckheere–Terpstra tests. All statistical analyses were performed using PASW Statistics 18 (SPSS Inc., Chicago, IL, USA), and *P* values of less than 0.05 were considered to be significant.

## Results

### Establishment and validation of neutralization assays based on the Ad4-Luc recombinant virus

A specific HAdV-4 neutralization assay was established based on a replication-competent Ad4-Luc recombinant virus that was constructed by replacing the E3 regions with a luciferase expression cassette.

To obtain an accurate readout of the neutralization assay, we first determined suitable amounts of the Ad4-Luc recombinant virus for detecting neutralizing antibodies. To this end, serial dilutions of the Ad4-Luc recombinant virus were added to 2 × 10^4^ A549 cells in 96-well plates, and cytopathic effects and luciferase expression were measured 24 h after infection (Fig. 1a, b). We observed that an IFU/cell ratio from 0.25 to 4 was optimal, as this range showed a good linear correlation between viral load and luciferase expression. At ratios lower than this range the luciferase assay value became unstable, while at higher ratios the linear relationship between luciferase expression and the viral load was lost due to the increase in cytopathy. Next, serial dilutions of the HAdV-4-positive serum samples were incubated with a fixed amount of Ad4-Luc at three IFU/cell ratios (0.5, 1, and 1.5) to calculate serum titers. The results showed no significant difference between the three groups (one-way ANOVA test, *P* value = 0.9; Fig. 1d), indicating that the method was highly stable. A representative neutralization curve of the serial dilutions of HAdV-4-positive sera incubated with Ad4-Luc at a fixed IFU/cell ratio of 1 is shown in Fig. 1c. To further verify the accuracy of this method, we tested 18 HAdV-4-positive serum samples by the luciferase expression inhibition and cytopathic effect assays simultaneously. Because high serum ratios (1:4 and 1:12) affected the growth of HEK293 cells, resulting in decreased cell viability in the cytopathic effect assay, only IC_50_ titers were obtained in this experiment. As expected, a good correlation in serum titers measured by the two methods was observed (Spearman correlation test, *P* value < 0.0001, Spearman *r* = 0.89). These results suggested that the adenovirus neutralization assays based on luciferase expression inhibition were stable and accurate.

### Study participants and characteristics

The demographics of all serum donors (*n* = 1013) are shown in Table 1. The participants were aged 18–65 (mean: 42.3 ± 12.2), 18–60 (mean: 41.8 ± 10.2), and 18–50 (mean: 32.4 ± 8.8) in Beijing, Jiangsu, and Freetown, respectively, including similar numbers of males and females. Both Beijing and Jiangsu represent the most densely populated regions in China, as does Freetown in Sierra Leone.

**Table 1:** Demographics of all serum sample donors (*n* = 1013)

Group	Beijing, *n* (%)	Jiangsu, *n* (%)	Freetown, *n* (%)	Total, *n* (%)
*Age (years)*				
≤30	48 (18.8%)	40 (15.4%)	244 (49.0%)	332 (32.8%)
31–40	69 (27.1%)	74 (28.5%)	146 (29.3%)	289 (28.5%)
41–50	63 (24.7%)	86 (33.1%)	108 (21.7%)	257 (25.4%)
>50	75 (29.4%)	60 (23.1%)	0 (0)	135 (13.3%)
*Gender*				
Male	120 (47.1%)	123 (47.3%)	268 (53.8%)	511 (50.4%)
Female	135 (52.9%)	137 (52.7%)	230 (46.2%)	502 (49.6%)
*Total*	255 (100%)	260 (100%)	498 (100%)	1013 (100%)

### Overall seroprevalence of HAdV-4 and HAdV-5 nAbs in healthy blood donors from China and Sierra Leone

**Fig. 2:**
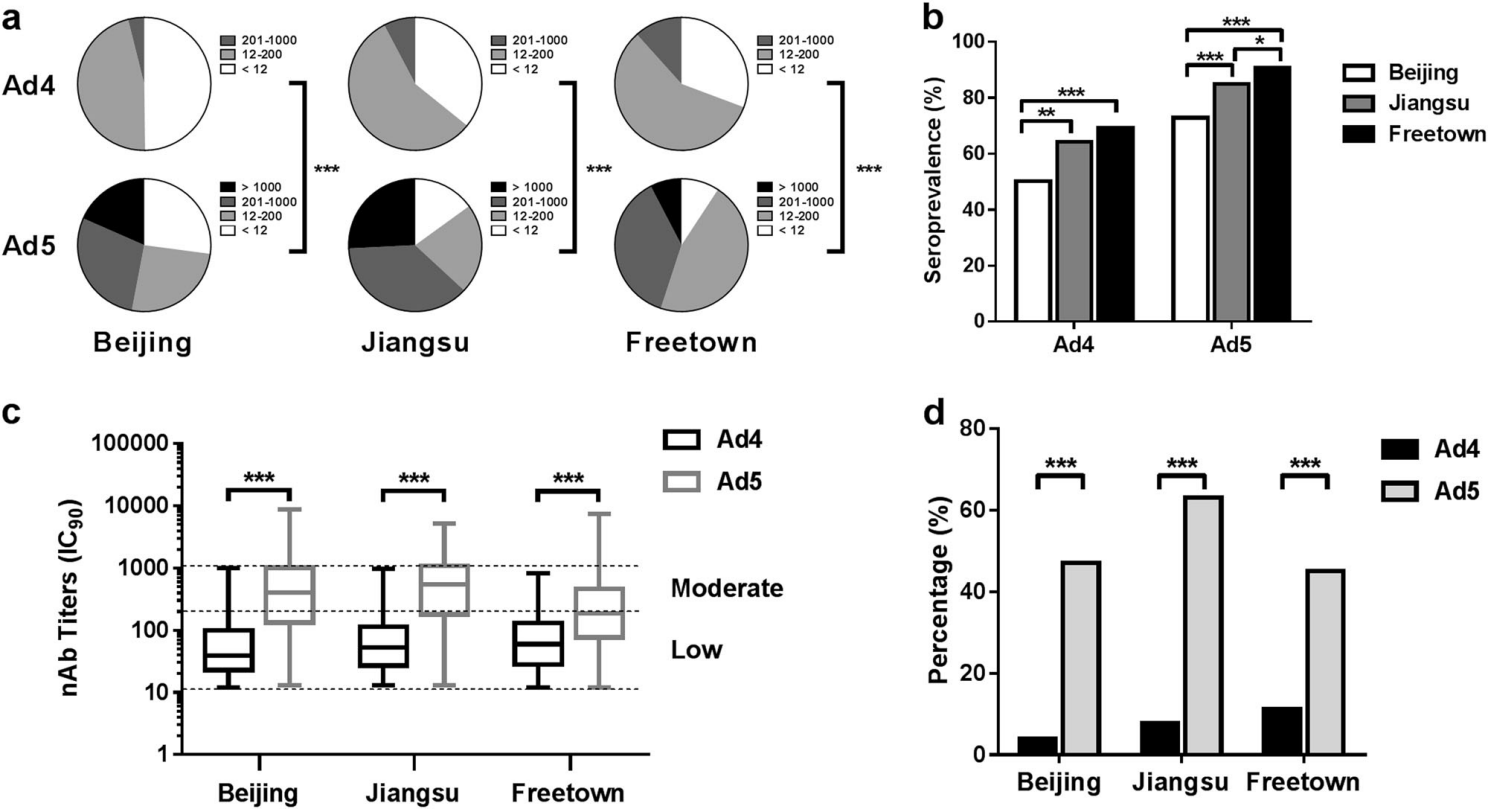
Seroprevalences of HAdV-4 and HAdV-5 nAbs in healthy blood donors in China (Beijing, *n* = 255, and Jiangsu, *n* = 260) and Sierra Leone (Freetown, *n* = 498). The nAb titers of HAdV-4 and HAdV-5 were tested using Ad4-Luc- and Ad5-Luc-based neutralization assays (IC_90_) and were categorized into four subgroups as follows: negative, <12; low, 12–200; moderate, 201–1000; and high, >1000. The nAb distributions (**a**) and the seropositive rates (**b**) of HAdV-4 and HAdV-5 in Beijing, Jiangsu, and Sierra Leone are shown. The data were analyzed by the Pearson chi-square test. **c** The difference in the nAb titers between HAdV-4- and HAdV-5-positive serum samples was analyzed by the Mann–Whitney test. **d** The percentages of serum samples with moderate or high levels of HAdV-4 and HAdV-5 nAbs are shown. The comparison was performed with the Pearson chi-square test. In all of the above comparison tests, a *P* value of <0.05 was considered to be significant; ^**^P value < 0.01, ^***^P value < 0.001

The nAb titers in the 1013 serum samples from the three different regions were determined based on the luciferase expression neutralization assay (Supplementary Table S1). The overall seropositive rates of HAdV-4 in Beijing, Jiangsu, and Sierra Leone were 50.2% (95% CI: 44.0–56.4%), 64.2% (95% CI: 58.4–70.1%), and 69.3% (95% CI: 65.2–73.3%), while for HAdV-5 these rates were 72.9% (95% CI: 67.5–78.4%), 85.0% (95% CI: 80.6–89.4%), and 90.8% (95% CI: 88.2–93.3%), respectively (Fig. 2a). The seropositive rates of HAdV-4 were notably much lower than those of HAdV-5 in all three locations (Pearson chi-square test, *P* value < 0.001). Interestingly, the HAdV-4 or HAdV-5 seropositive rates in different regions varied, with highest rates observed in Sierra Leone, followed by Jiangsu, while the lowest rates were observed in Beijing (Pearson chi-square test) (Fig. 2b).

Although the HAdV-4 seropositive rates exceeded 50%, the nAb titers of over 80% of the HAdV-4-positive serum samples ranged from 12 to 200 (Fig. 2c). However, approximately 50–75% of the HAdV-5-positive serum samples had nAb titers of over 200. The geometric mean titers of the HAdV-4 nAbs were 48.9 (95% CI: 41.5–57.6), 58.9 (95% CI: 51.1–68.0), and 65.0 (95% CI: 58.4–72.4) for the HAdV-4 seropositive samples from Beijing, Jiangsu, and Freetown, respectively. And the geometric mean titers of the HAdV-5 nAbs for the HAdV-5 seropositive samples from those regions were 333.5 (95% CI: 273.4–406.8), 429.5 (95% CI: 361.8–509.8), and 186.4 (95% CI: 165.8–209.5), respectively. The HAdV-4 nAb titers were significantly lower than those for HAdV-5 in seropositive samples (Mann–Whitney test, *P* value < 0.001). The percentage of serum samples with moderate or high nAb levels from Beijing, Jiangsu, and Freetown was 3.9% (95% CI: 1.5–6.3%), 7.7% (95% CI: 4.4–11.0%), and 11.6% (95% CI: 8.8–14.5%) for HAdV-4 and 47.1% (95% CI: 40.9–53.2%), 63.1% (95% CI: 57.2–69.0%) and 45.0% (95% CI: 40.6–49.4%) for HAdV-5, respectively (Fig. 2d). The results indicated that the seroprevalence of HAdV-4 nAbs was significantly lower than that of HAdV-5 in all three locations (Pearson chi-square test).

### Distribution of HAdV-4 and HAdV-5 nAbs in different gender groups

**Fig. 3:**
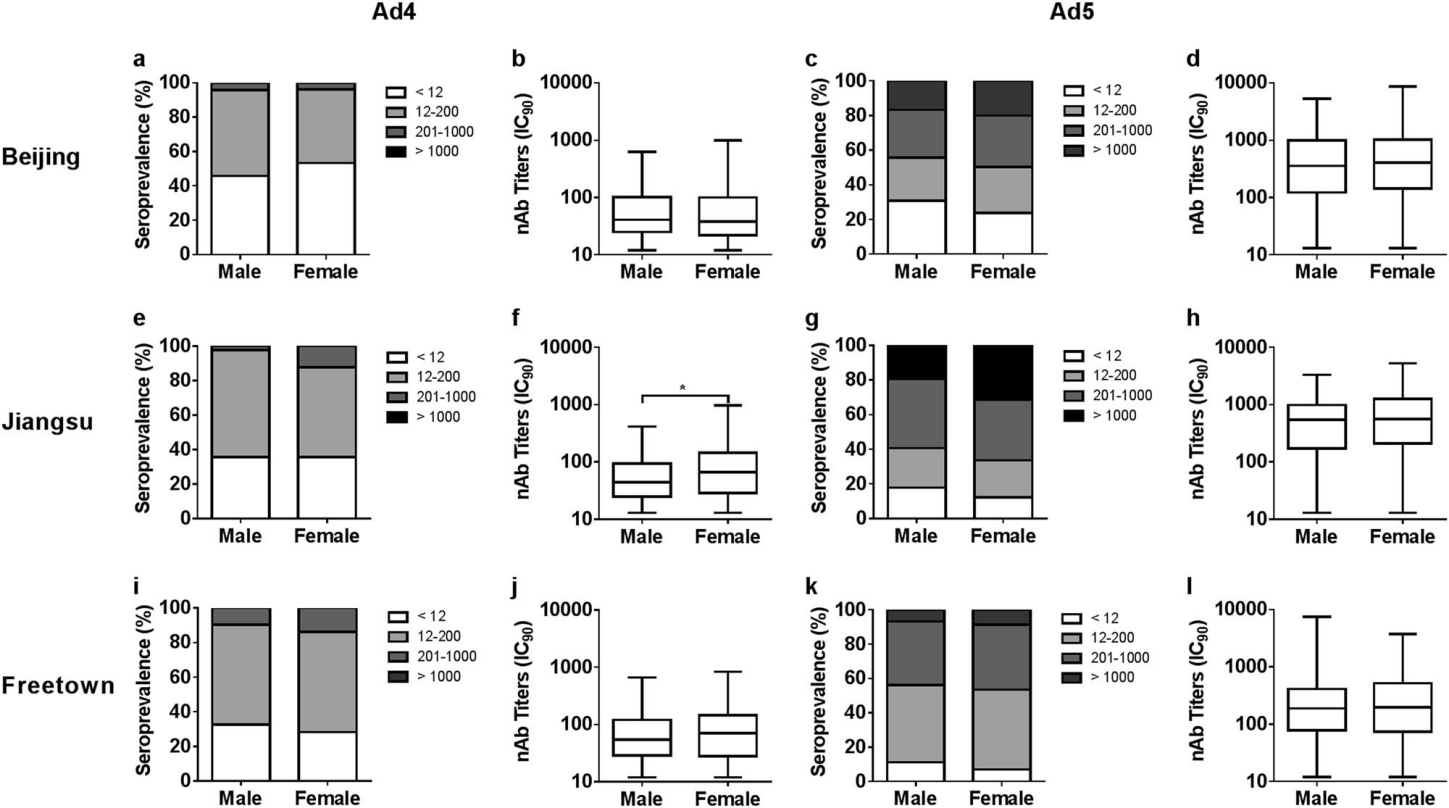
Distributions of HAdV-4 and HAdV-5 nAbs between gender groups in different regions of China and Sierra Leone. The distributions of HAdV-4 and HAdV-5 nAbs between males and females in Beijing (**a**, **c**), Jiangsu (**e**, **g**) and Freetown (**i**, **k**) are shown. The comparisons of adenovirus seropositive rates between gender groups were analyzed by Pearson chi-square tests. The HAdV-4 and HAdV-5 nAb titers between males and females with detectable nAbs from Beijing (**b**, **d**), Jiangsu (**f**, **h**), and Freetown (**j**, **l**) are shown. The data were analyzed by Mann–Whitney tests. In all the above comparison tests, a *P* value of <0.05 was considered significant

The results showed no significant differences in HAdV-4 seropositive rates between males and females in Beijing, Jiangsu, and Freetown (Pearson chi-square tests, *P* value = 0.232, 0.999, and 0.270; Fig. 3a, e, i, respectively; Supplementary Table S2), similar to that observed for the HAdV-5 seropositive rates (Pearson chi-square tests, *P* value = 0.201, 0.217, and 0.103; Fig. 3c, g, k, respectively). The HAdV-4 nAb titers were comparable between males and females in Beijing and Freetown (Mann–Whitney tests, *P* value = 0.393 and 0.153; Fig. 3b, j, respectively), whereas higher HAdV-4 nAb titers were detected in females than in males in Jiangsu (Mann–Whitney test, *P* value = 0.013; Fig. 3f).

In addition, no significant differences in HAdV-5 nAb titers were detected between gender groups in Beijing, Jiangsu, and Freetown (Mann–Whitney tests, *P* value = 0.896, 0.356, and 0.504; Fig. 3d, h, l, respectively). Comparisons of the adenovirus seropositive rates and nAb titers between gender groups suggested that the sensitivity of men and women to infection by HAdV-4 or HAdV-5 is consistent and that the same level of nAbs could be generated by males and females after infection with HAdV-4 or HAdV-5.

### Distribution of HAdV-4 and HAdV-5 nAbs in different age groups

**Fig. 4:**
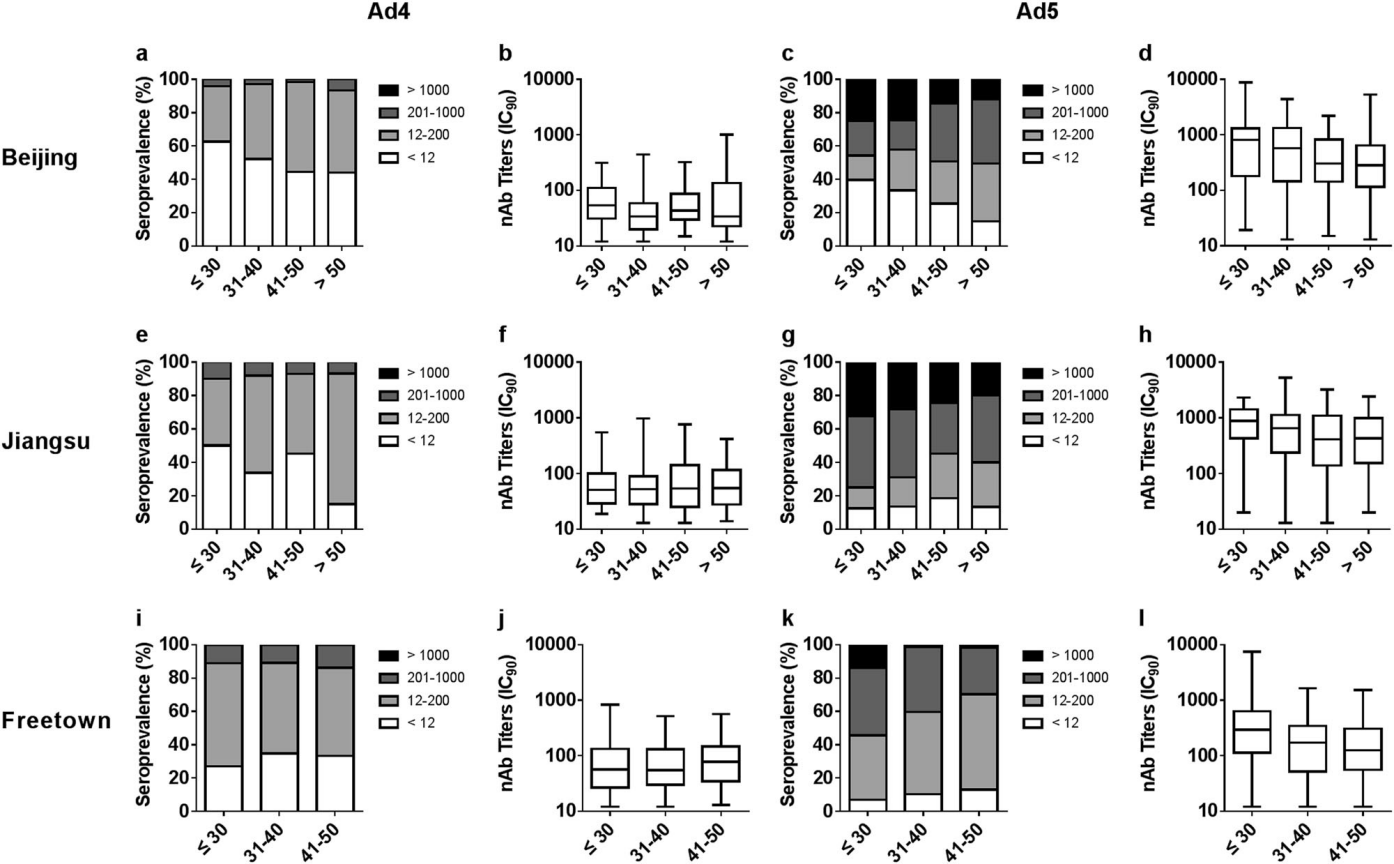
Distributions of HAdV-4 and HAdV-5 nAbs in different age groups and regions of China and Sierra Leone. The distribution of HAdV-4 and HAdV-5 nAbs in age groups in Beijing (**a**, **c**), Jiangsu (**e**, **g**), and Freetown (**i**, **k**) are shown. The trends in adenovirus seropositive rate versus age were analyzed by Cochran–Armitage trend tests. The titers of HAdV-4 and HAdV-5 nAbs in seropositive donor age groups in Beijing (**b**, **d**), Jiangsu (**f**, **h**), and Freetown (**j**, **l**) are shown. The data were analyzed by Jonckheere–Terpstra tests

We next examined differences in the seropositive rates and nAb levels among the different age groups (Supplementary Tables S3 and S4). For different adenoviruses and in different regions, the distribution of nAbs versus age varied greatly. The seropositive rates of HAdV-4 increased with age in Beijing and Jiangsu, China (Cochran–Armitage trend tests, *P* value = 0.037 and 0.004; Fig. 4a, e), but not in Freetown, Sierra Leone (Cochran–Armitage trend tests, *P* value = 0.15; Fig. 4i). Although the trends in HAdV-4 seroprevalence were different in China and Sierra Leone, the HAdV-4 nAb levels were consistent among the different age groups in different regions (Jonckheere–Terpstra tests; Fig. 4b, f, j).

For HAdV-5, the trends in seropositive rates among the age groups in Beijing, China, were similar to those of HAdV-4 in that increased HAdV-5 seroprevalence versus age was observed (Cochran–Armitage trend tests, *P* value < 0.001; Fig. 4c). Interestingly, no differences in HAdV-5 seroprevalence among the age groups in Jiangsu and Freetown were observed (Cochran–Armitage trend tests; Fig. 4g, k), but the percentage of serum samples with moderate or high nAb titers decreased with age (Cochran–Armitage trend tests, *P* value = 0.044 in Jiangsu and <0.001 in Freetown). In addition, the HAdV-5 nAb levels in seropositive donors definitely decreased with age in all regions (Jonckheere–Terpstra test, *P* value = 0.013 and 0.019 in Beijing and Jiangsu, respectively, <0.001 in Freetown; Fig. 4d, h, l).

### Cross-neutralization reaction analysis between HAdV-4 and HAdV-5 nAbs

**Fig. 5:**
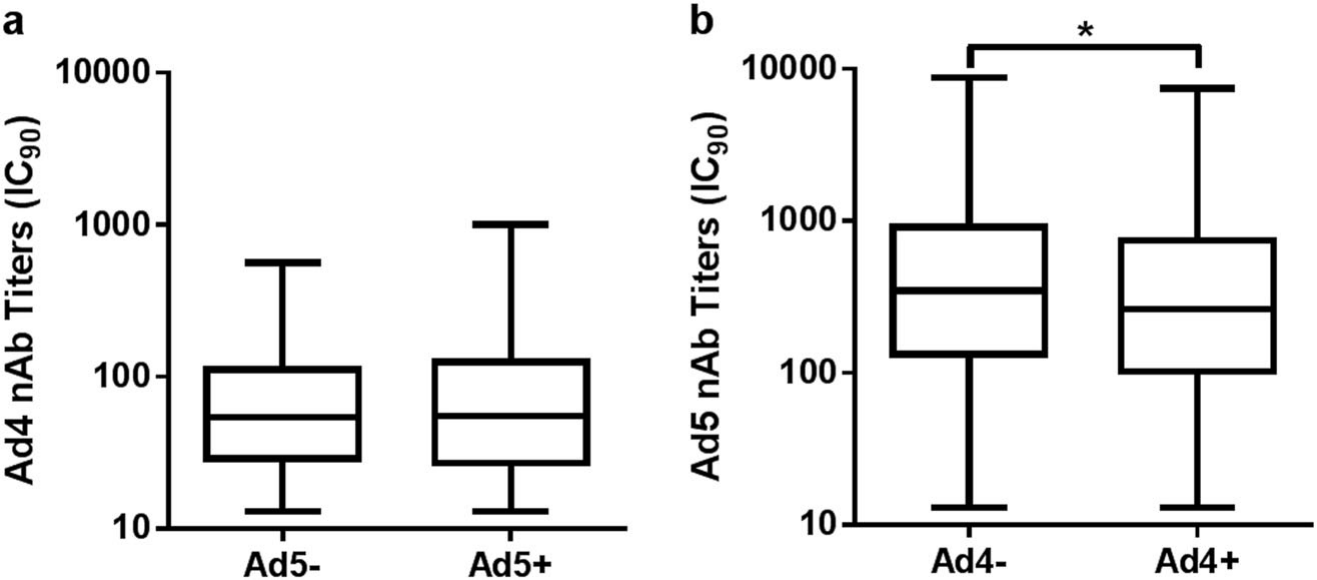
The distribution of HAdV-4 or HAdV-5 nAbs in single- and double-positive blood donors. The nAb levels of HAdV-5 (**a**) or HAdV-4 (**b**) in single- and double-positive blood donors are shown. The comparison was analyzed by Mann–Whitney tests, and a *P* value of <0.05 was considered significant

No differences in HAdV-4 seroprevalence were detected between the HAdV-5 seronegative and HAdV-5 seropositive samples, and the same trend was observed for HAdV-5 seroprevalence in the HAdV-4 seronegative and HAdV-4 seropositive groups (chi-square test, *P* = 0.967; Table 2). However, the HAdV-5 nAb titers in the HAdV-4 seropositive samples were significantly lower than those that were HAdV-4 seronegative (Mann–Whitney test, *P* = 0.011; Fig. 5a), while the level of HAdV-4 nAbs was not affected by HAdV-5 seroprevalence (Mann–Whitney test, *P* = 0.755; Fig. 5b). These results suggested that there was scarcely any cross-neutralization reaction between HAdV-4 and HAdV-5, and the probability of HAdV-4 or HAdV-5 infection was not affected by the presence of nAbs of the other serotype, although HAdV-5 nAbs were slightly reduced in HAdV-4 seropositive donors.

**Table 2:** Cross-neutralization reaction analysis between HAdV-4 and HAdV-5 nAbs

	HAdV-4 seronegative^a^	HAdV-4 seropositive^b^	HAdV-4 seroprevalence^c^ (%)
HAdV-5 seronegative^a^	57	97	97/154 (63.0)
HAdV-5 seropositive^b^	316	543	543/859 (63.2)
HAdV-5 seroprevalence (%)^d^	316/373 (84.7)	543/640 (84.8)	

^a^The number of HAdV-4- or HAdV-5-negative serum samples

^b^The number of HAdV-4- or HAdV-5-positive serum samples

^c^The rate of HAdV-4-positive results in HAdV-5-negative or HAdV-5-positive serum samples

^d^The rate of HAdV-5-positive results in HAdV-4-negative or HAdV-4-positive serum samples

Correlation was analyzed by chi-square test, *P* = 0.957

## Discussion

According to previous epidemiological and clinical studies of HAdV-4 infection among US military trainees, HAdV-4 specific nAbs were observed in 97% of the recruits naturally infected with the HAdV-4 virus, and HAdV-4 could be isolated from 92% of the patients with increased HAdV-4 specific nAbs^12^. These findings demonstrated that natural HAdV-4 infection can give rise to specific antibodies, which is also an important indicator of infection. In the present study, high-seropositive rates of HAdV-5 nAbs were observed, similar to those reported in other studies^20–25^. To our surprise, over 50% of participants in China and nearly 70% of donors in Sierra Leone possessed detectable nAbs against HAdV-4, even though few HAdV-4 infection cases in these areas were reported. These results suggested that HAdV-4 may be an underestimated pathogen among adults in China and parts of Africa.

A neutralization assay based on Ad5-Luc is commonly used to assess the prevalence of HAdV-5 nAbs^20,23,24^, and this assay is reported to be more sensitive and objective than the previously used CPE-based assays^19^. In this study, an HAdV-4 neutralization assay was developed following the example of HAdV-5, except that both the E1 and E3 regions were deleted from the adenovirus genomes of HAdV-5, while only the E3 region was deleted in HAdV-4, resulting in replication-competent Ad4-Luc and replication-defective Ad5-Luc, respectively. The establishment of this type of method is based on the linear relationship between the logarithm of viral load and the logarithmic value of luciferase expression, as well as the “S-type distribution” between luciferase expression and the logarithms of serum serial dilutions when quantities of the virus are fixed (Fig. 1)^19,26^. The method remained accurate when amounts of recombinant virus within a certain range were added, and the results were consistent with those of traditional CPE-based neutralization assays. Considering the difficulties and time required for isolating and identifying HAdV-4 from limited samples, such as throat swabs and stool^27^, this method provides a more convenient (serum samples) and sensitive (antibody response) method of HAdV-4 surveillance. Moreover, this method could also be used to assess immune response and efficacy during the development of HAdV-4 vaccines or HAdV-4-based recombinant vaccines when necessary.

The observed prevalence of naturally occurring nAbs to HAdV-4 was lower than that of HAdV-5 nAbs in China and Sierra Leone (Fig. 2). The same trend was also observed in the US, where the observed seropositive rate was 36.0% for HAdV-4^28^ and 69.1% for HAdV-5^22^. The prevalence of HAdV-4 and HAdV-5 nAbs varied by geographic location and appeared to be significantly higher in West Africa than in China and in Jiangsu than in Beijing, the capital city of China. Geographical differences have also been observed in other epidemiological studies of HAdV-4 and HAdV-5^21,22,24,25^, and these variations may be attributable to climate (higher nAbs among populations who have spent a great deal of time in tropical regions), crowding (high-population density may facilitate transmission), or sanitary conditions^12,15,22,29^. Thus, these results revealed a greater risk of adenovirus infection in developing countries due to heat, crowding, and poor sanitation.

Although more than 50% of serum donors were seropositive for HAdV-4 nAbs in China and Africa, the nAb titers of seropositive samples were almost uniformly distributed at low levels (≤200), and serum donors with moderate to high levels (>200) of nAbs accounted for 3.9, 7.7, and 11.7% in Beijing, Jiangsu, and Freetown, respectively. Notably, only moderate to high levels of nAbs are considered to suppress the immunogenicity of adenovirus-vectored recombinant vaccines^30–32^, suggesting that risks of HAdV-4 infection among seropositive adults should not be ignored. These results also indicate that more attention should be paid to improving the level of nAbs induced by HAdV-4 vaccines developed in the future. However, this circumstance benefits the development of HAdV-4-based vaccines, as most people have a lower baseline level of the neutralizing antibody titers. Considering that the HAdV-4 oral vaccine has been safely used in the American military for a long time, and no cross-neutralization reaction has been observed with HAdV-5 (Table 2), live oral vaccines based on replication-competent HAdV-4 vectors have a large potential for wide application.

Similar distributions in seropositive rates and nAb titers of HAdV-4 or HAdV-5 between gender groups were observed (Fig. 3), in accordance with the results observed for other adenovirus types^4,29^. These results suggest that the sensitivity of men and women to infection by HAdV-4 or HAdV-5 was consistent and that the same level of nAbs could be generated by males and females after infection with HAdV-4 or HAdV-5. We also observed that the seropositive rates of HAdV-4 in young adults were low and increased with age (Fig. 4) in China, with the same trend observed for HAdV-5 in Beijing, China. Previous surveys have reported that the seropositive rates of HAdV-5 and those other HAdVs, such as HAdV-14 and HAdV-55, increase with age^4,24^. However, no significant difference was observed among age groups in HAdV-4 seroprevalence in Africa or in HAdV-5 seroprevalence in Jiangsu and Africa. For all three of these combinations, the seropositive rates in the younger age groups were above 70%, perhaps too high to show a significant increase. This phenomenon was also observed in other investigations of HAdV-5 and is defined as a “peak-and-stable” type^23,24^, although the reason for this phenomenon has not been elucidated. It is well-known that neutralizing antibodies stimulated by adenoviruses or adenovirus-based vaccines can persist for a long time^25,28,33,34^. However, a dynamic study of HAdV-5 nAbs indicated that some exceptions exist, as some individuals who were seropositive and had high levels of nAbs during the first visit rapidly became seronegative^25^. In our previous clinical studies on the HAdV-5-based Ebola vaccine^26,30^, a low proportion of exceptions were also observed, where individuals whose nAbs against HAdV-5 became seropositive after immunization with HAdV-5-based vaccines but decreased rapidly to their original levels within a few months (data not shown). The reason for this type of unusual humoral immune response remains unclear.

In contrast, the trend in adenovirus seroprevalence among age groups may be different from the trend in antibody titers. In this study, HAdV-4 nAb levels in seropositive donors were consistent among different age groups for all specific regions, whereas they decreased with age for HAdV-5. Such a phenotype in nAbs of HAdV-4, the only human virus member of species E, has not been previously reported, whereas the same phenotype for HAdV-5, which belongs to species C, has been reported in previous studies^22^. However, an increase with age in nAbs of HAdV-14 and HAdV-55, which belong to species B, was observed in another investigation^4^. These results indicate a significant difference in age-dependent nAb response among different species of HAdVs. Our findings provide valuable insight into the humoral immune responses to different types of adenoviruses and are an important reference for vaccine design in clinical trials. The stable and low HAdV-4 nAb response in different age groups may indicate that postinfection morbidity among older individuals is the same as that in young populations, because they tend to produce the same level of nAbs. As expected, cases of HAdV-4 infection were observed more frequently in adults in a retrospective investigation^35^. Similarly, recent HAdV-4 infection cases have primarily occurred in adults during outbreaks in the US since 2011^22^. Notably, an outbreak of acute respiratory disease due to HAdV-4 and a resulting increase in mortality occurred in a long-term care facility for the elderly. Thus, surveillance for HAdV-4 infection should include populations of all ages, not only children.

In summary, we established a convenient neutralization assay based on replication-competent Ad4-Luc virus and investigated the seroprevalence of HAdV-4 nAbs in civilians from China and from Sierra Leone, West Africa. The high seroprevalence and the low level of HAdV-4 neutralizing antibodies predicted the potential risk of disease outbreaks in China and West Africa. Our results highlights the importance of HAdV-4 surveillance and provides useful insights for the future development of HAdV-4 or HAdV-4 vector vaccines.

## Electronic supplementary material

Supplementary Table S1

Supplementary Table S2

Supplementary Table S3

Supplementary Table S4

## References

[1] Thounaojam AD, Balakrishnan A, Mun AB (2016). Detection and molecular typing of human adenoviruses associated with respiratory illnesses in Kerala. Jpn. J. Infect. Dis..

[2] Heo JY (2018). Molecular epidemiology of human adenovirus-associated febrile respiratory illness in soldiers, South Korea. Emerg. Infect. Dis..

[3] Ismail AM (2017). Adenoviromics: mining the human adenovirus species D genome. Front. Microbiol..

[4] Zheng X (2017). Seroprevalence of neutralizing antibodies against adenovirus type 14 and 55 in healthy adults in Southern China. Emerg Microbes Infect.

[5] Wang YF (2016). Molecular epidemiology and clinical manifestations of adenovirus respiratory infections in Taiwanese Children. Medicine.

[6] Li QG, Zheng QJ, Liu YH, Wadell G (2001). Molecular epidemiology of adenovirus types 3 and 7 isolated from children with pneumonia in Beijing. J. Med. Virol..

[7] Hong JY (2001). Lower respiratory tract infections due to adenovirus in hospitalized Korean children: epidemiology, clinical features, and prognosis. Clin. Infect. Dis..

[8] Mandelboim M, Dror P, Azar R, Bromberg M, Mendelson E (2011). Adenovirus infections in hospitalized patients in Israel: epidemiology and molecular characterization. J. Clin. Microbiol..

[9] Gray GC (2007). Genotype prevalence and risk factors for severe clinical adenovirus infection, United States 2004–2006. Clin. Infect. Dis..

[10] Lu QB (2014). Epidemiology of human adenovirus and molecular characterization of human adenovirus 55 in China, 2009–2012. Influenza Other Respir. Virus..

[11] Li X (2014). An outbreak of acute respiratory disease in China caused by human adenovirus type B55 in a physical training facility. Int. J. Infect. Dis..

[12] Kolavic-Gray SA (2002). Large epidemic of adenovirus type 4 infection among military trainees: epidemiological, clinical, and laboratory studies. Clin. Infect. Dis..

[13] Gopalkrishna V, Ganorkar NN, Patil PR (2016). Identification and molecular characterization of adenovirus types (HAdV-8, HAdV-37, HAdV-4, HAdV-3) in an epidemic of keratoconjunctivitis occurred in Pune, Maharashtra, Western India. J. Med. Virol..

[14] Cheng JL (2017). Risk factor analysis and molecular epidemiology of respiratory adenovirus infections among children in northern Taiwan, 2009–2013. J. Microbiol. Immunol. Infect..

[15] Kajon AE (2018). Adenovirus type 4 respiratory infections among civilian adults, Northeastern United States, 2011–2015. Emerg. Infect. Dis..

[16] Narra R (2016). Acute respiratory distress syndrome in adenovirus type 4 pneumonia: a case report. J. Clin. Virol..

[17] Kalimuddin, S. et al. A report of adult human adenovirus infections in a tertiary hospital. *Open Forum Infect. Dis.* **4**, 10.1093/ofid/ofx053 (2017). 10.1093/ofid/ofx053 PMC5419200 28491891

[18] Weaver EA (2013). Vaccines within vaccines. Hum. Vaccin. Immunother..

[19] Sprangers MC (2003). Quantifying adenovirus-neutralizing antibodies by luciferase transgene detection: addressing preexisting immunity to vaccine and gene therapy vectors. J. Clin. Microbiol..

[20] Abbink P (2007). Comparative seroprevalence and immunogenicity of six rare serotype recombinant adenovirus vaccine vectors from subgroups B and D. J. Virol..

[21] Nwanegbo E (2004). Prevalence of neutralizing antibodies to adenoviral serotypes 5 and 35 in the adult populations of the Gambia, South Africa, and the United States. Clin. Vacc. Immunol..

[22] Mast TC (2010). International epidemiology of human pre-existing adenovirus (Ad) type-5, type-6, type-26 and type-36 neutralizing antibodies: correlates of high Ad5 titers and implications for potential HIV vaccine trials. Vaccine.

[23] Huang D (2015). The sero-prevalence of anti-adenovirus 5 neutralizing antibodies is independent of a chronic hepatitis B carrier state in China. Arch. Virol..

[24] Yu B (2012). A serological survey of human adenovirus serotype 2 and 5 circulating pediatric populations in Changchun, China, 2011. Virol. J..

[25] Sun C (2011). Epidemiology of adenovirus type 5 neutralizing antibodies in healthy people and AIDS patients in Guangzhou, southern China. Vaccine.

[26] Aste-Amézaga M (2004). Quantitative adenovirus neutralization assays based on the secreted alkaline phosphatase reporter gene: application in epidemiologic studies and in the design of adenovector vaccines. Hum. Gene Ther..

[27] Wigand R (1987). Pitfalls in the identification of adenoviruses. J. Virol. Methods.

[28] Kuschner RA (2013). A phase 3, randomized, double-blind, placebo-controlled study of the safety and efficacy of the live, oral adenovirus type 4 and type 7 vaccine, in U.S. military recruits. Vaccine.

[29] Yang WX (2016). Prevalence of serum neutralizing antibodies to adenovirus type 5 (Ad5) and 41 (Ad41) in children is associated with age and sanitary conditions. Vaccine.

[30] Appaiahgari MB, Saini M, Rauthan M, Jyoti, Vrati S (2006). Immunization with recombinant adenovirus synthesizing the secretory form of Japanese encephalitis virus envelope protein protects adenovirus-exposed mice against lethal encephalitis. Microbes Infect..

[31] Zhu FC (2015). Safety and immunogenicity of a novel recombinant adenovirus type-5 vector-based Ebola vaccine in healthy adults in China: preliminary report of a randomised, double-blind, placebo-controlled, phase 1 trial. Lancet.

[32] Feng Y (2018). An adenovirus serotype 2-vectored ebolavirus vaccine generates robust antibody and cell-mediated immune responses in mice and *Rhesus macaques*. Emerg. Microbes Infect..

[33] Li JX (2017). Immunity duration of a recombinant adenovirus type-5 vector-based Ebola vaccine and a homologous prime-boost immunisation in healthy adults in China: final report of a randomised, double-blind, placebo-controlled, phase 1 trial. Lancet Glob. Health.

[34] Zhu FC (2017). Safety and immunogenicity of a recombinant adenovirus type-5 vector-based Ebola vaccine in healthy adults in Sierra Leone: a single-centre, randomised, double-blind, placebo-controlled, phase 2 trial. Lancet.

[35] Schmttz H, Wigand R, Heinrich W (1983). Worldwide epidemiology of human adenovirus infections. Am. J. Epidemiol..

