# Supplementary material for: Seroepidemiological investigation of HAdV-4 infection among healthy adults in China and in Sierra Leone, West Africa
Source: Emerg Microbes Infect. 2018 Dec 5;7:200. doi: 10.1038/s41426-018-0206-y (PMC6279822; doi:10.1038/s41426-018-0206-y)
Supplement: Supplementary file 1 — Supplementary Table S1 [file 41426_2018_206_MOESM1_ESM.docx]

**Supplementary Table S1**

**HAdV-4 and HAdV-5 nAb seroprevalence in healthy adults from China and Sierra Leone**

|  | **HAdV-4 neutralizing antibody titer [n (%)]*^a^*** | | | | **Total [n (%)]** |
| --- | --- | --- | --- | --- | --- |
|  | **<12** | **12-200** | **201-1000** | **>1000** |  |
| **HAdV-4** |  |  |  |  |  |
| Beijing, China | 127 (49.8) | 118 (46.3) | 10 (3.9) | 0 (0) | 255 (100) |
| Jiangsu, China | 93 (35.8) | 147 (56.5) | 20 (7.7) | 0 (0) | 260 (100) |
| Freetown, Sierra Leone | 153 (30.7) | 287 (57.6) | 58 (11.6) | 0 (0) | 498 (100) |
| China (Beijing & Jiangsu) | 220 (42.7) | 265 (51.5) | 30 (5.8) | 0 (0) | 515 (100) |
| Overall | 373 (36.8) | 552 (54.5) | 88 (8.7) | 0 (0) | 1013 (100) |
| **HAdV-5** |  |  |  |  |  |
| Beijing, China | 69 (27.1) | 66 (25.9) | 73 (28.6) | 47 (18.4) | 255 (100) |
| Jiangsu, China | 39 (15.0) | 57 (21.9) | 97 (37.3) | 67 (25.8) | 260 (100) |
| Freetown, Sierra Leone | 46 (9.2) | 228 (45.8) | 186 (37.3) | 38 (7.6) | 498 (100) |
| China (Beijing & Jiangsu) | 108 (21.0) | 123 (23.9) | 170 (33.0) | 114 (22.1) | 515 (100) |
| Overall | 154 (15.2) | 351 (34.6) | 356 (35.1) | 152 (15.0) | 1013 (100) |

*^a^* The absolute number and the percentage in the respective subgroups were shown.
