# Supplementary material for: Seroepidemiological investigation of HAdV-4 infection among healthy adults in China and in Sierra Leone, West Africa
Source: Emerg Microbes Infect. 2018 Dec 5;7:200. doi: 10.1038/s41426-018-0206-y (PMC6279822; doi:10.1038/s41426-018-0206-y)
Supplement: Supplementary file 2 — Supplementary Table S2 [file 41426_2018_206_MOESM2_ESM.docx]

**Supplementary Table S2**

**HAdV-4 and HAdV-5 nAb seroprevalence between gender groups from China and Sierra Leone**

|  | **Beijing, China** | | **Jiangsu, China** | | **Freetown, Sierra Leone** | | **Overall** | |
| --- | --- | --- | --- | --- | --- | --- | --- | --- |
|  | Male | Female | Male | Female | Male | Female | Male | Female |
| **HAdV-4*^a^*** |  |  |  |  |  |  |  |  |
| <12 | 55 (45.8) | 72 (53.3) | 44 (35.8) | 49 (35.8) | 88 (32.8) | 65 (28.3) | 187 (36.6) | 186 (37.1) |
| 12-200 | 60 (50) | 58 (43.0) | 76 (61.8) | 71 (51.8) | 154 (57.5) | 133 (57.8) | 290 (56.8) | 262 (52.2) |
| 201-1000 | 5 (4.2) | 5 (3.7) | 3 (2.4) | 17 (12.4) | 26 (9.7) | 32 (13.9) | 34 (6.7) | 54 (10.8) |
| >1000 | 0 (0) | 0 (0) | 0 (0) | 0 (0) | 0 (0) | 0 (0) | 0 (0) | 0 (0) |
| Total | 120 (100) | 135 (100) | 123 (100) | 137 (100) | 268 (100) | 230 (100) | 511 (100) | 502 (100) |
| **HAdV-5 *^b^*** |  |  |  |  |  |  |  |  |
| <12 | 37 (30.8) | 32 (23.7) | 22 (17.9) | 17 (12.4) | 30 (11.2) | 16 (7.0) | 89 (17.4) | 65 (12.9) |
| 12-200 | 30 (25.0) | 36 (26.7) | 28 (22.8) | 29 (21.2) | 121 (45.1) | 107 (46.5) | 179 (35.0) | 172 (34.3) |
| 201-1000 | 33 (27.5) | 40 (29.6) | 49 (39.8) | 48 (35.0) | 99 (36.9) | 87 (37.8) | 181 (35.4) | 175 (34.9) |
| >1000 | 20 (16.7) | 27 (20.0) | 24 (19.5) | 43 (31.4) | 18 (6.7) | 20 (8.7) | 62 (12.1) | 90 (17.9) |
| Total | 120 (100) | 135 (100) | 123 (100) | 137 (100) | 268 (100) | 230 (100) | 511 (100) | 502 (100) |

*^a^* The absolute number and the percentage of HAdV-4 nAbs in each subgroup.

*^b^* The absolute number and the percentage of HAdV-5 nAbs in each subgroup.
