# Supplementary material for: Seroepidemiological investigation of HAdV-4 infection among healthy adults in China and in Sierra Leone, West Africa
Source: Emerg Microbes Infect. 2018 Dec 5;7:200. doi: 10.1038/s41426-018-0206-y (PMC6279822; doi:10.1038/s41426-018-0206-y)
Supplement: Supplementary file 3 — Supplementary Table S3 [file 41426_2018_206_MOESM3_ESM.docx]

**Supplementary Table S3**

**HAdV-4 nAb seroprevalence in different age groups from China and Sierra Leone**

|  | **HAdV-4 neutralizing antibody titer [n (%)]***^a^* | | | | **Total [n (%)]** |
| --- | --- | --- | --- | --- | --- |
|  | **<12** | **12-200** | **201-1000** | **>1000** |  |
| **Beijing, China***^b^* |  |  |  |  |  |
| <=30 | 30 (62.5) | 16 (33.3) | 2 (4.2) | 0 (0) | 48 (100.0) |
| 31-40 | 36 (52.2) | 31 (44.9) | 2 (2.9) | 0 (0) | 69 (100.0) |
| 41-50 | 28 (44.4) | 34 (54.0) | 1 (1.6) | 0 (0) | 63 (100.0) |
| >50 | 33 (44.0) | 37 (49.3) | 5 (6.7) | 0 (0) | 75 (100.0) |
| Total | 127 (49.8) | 118 (46.3) | 10 (3.9) | 0 (0) | 255 (100.0) |
| **Jiangsu, China***^c^* |  |  |  |  |  |
| <=30 | 20 (50.0) | 16 (40.0) | 4 (10.0) | 0 (0) | 40 (100.0) |
| 31-40 | 25 (33.8) | 43 (58.1) | 6 (8.1) | 0 (0) | 74 (100.0) |
| 41-50 | 39 (45.3) | 41 (47.7) | 6 (7.0) | 0 (0) | 86 (100.0) |
| >50 | 9 (15.0) | 47 (78.3) | 4 (6.7) | 0 (0) | 60 (100.0) |
| Total | 93 (35.8) | 147 (56.5) | 20 (7.7) | 0 (0) | 260 (100.0) |
| **Freetown, Sierra Leone***^d^* | |  |  |  |  |
| <=30 | 66 (27.0) | 151 (61.9) | 27 (11.1) | 0 (0) | 244 (100.0) |
| 31-40 | 51 (34.9) | 79 (54.1) | 16 (11.0) | 0 (0) | 146 (100.0) |
| 41-50 | 36 (33.3) | 57 (52.8) | 15 (13.9) | 0 (0) | 108 (100.0) |
| Total | 153 (30.7) | 287 (57.6) | 58 (11.6) | 0 (0) | 498 (100.0) |
| **Overall** |  |  |  |  |  |
| <=30 | 116 (34.9) | 183 (55.1) | 33 (9.9) | 0 (0) | 332 (100.0) |
| 31-40 | 112 (38.8) | 153 (52.9) | 24 (8.3) | 0 (0) | 289 (100.0) |
| 41-50 | 103 (40.1) | 132 (51.4) | 22 (8.6) | 0 (0) | 257 (100.0) |
| >50 | 42 (31.1) | 84 (62.2) | 9 (6.7) | 0 (0) | 135 (100.0) |
| Total | 373 (36.8) | 552 (54.5) | 88 (8.7) | 0 (0) | 1013 (100.0) |

*^a^* The absolute number and the percentage of HAdV-4 nAbs in each subgroup.

*^b^* The ages of serum donors from Beijing, China, ranged from 18 to 65 years old.

*^c^* The ages of serum donors from Jiangsu, China, ranged from 18 to 60 years old.

*^d^* The ages of serum donors from Freetown, Sierra Leone, ranged from 18 to 50 years old.
