# Supplementary material for: Seroepidemiological investigation of HAdV-4 infection among healthy adults in China and in Sierra Leone, West Africa
Source: Emerg Microbes Infect. 2018 Dec 5;7:200. doi: 10.1038/s41426-018-0206-y (PMC6279822; doi:10.1038/s41426-018-0206-y)
Supplement: Supplementary file 4 — Supplementary Table S4 [file 41426_2018_206_MOESM4_ESM.docx]

**Supplementary Table S4**

**HAdV-5 nAb seroprevalence in different age groups from China and Sierra Leone**

|  | **HAdV-5 neutralizing antibody titer [n (%)]***^a^* | | | | **Total [n (%)]** |
| --- | --- | --- | --- | --- | --- |
|  | **<12** | **12-200** | **201-1000** | **>1000** |  |
| **Beijing, China***^b^* |  |  |  |  |  |
| <=30 | 19 (39.6) | 7 (14.6) | 10 (20.8) | 12 (25.0) | 48 (100.0) |
| 31-40 | 23 (33.3) | 17 (24.6) | 12 (17.4) | 17 (24.6) | 69 (100.0) |
| 41-50 | 16 (25.4) | 16 (25.4) | 22 (34.9) | 9 (14.3) | 63 (100.0) |
| >50 | 11 (14.7) | 26 (34.7) | 29 (38.7) | 9 (12.0) | 75 (100.0) |
| Total | 69 (27.1) | 66 (25.9) | 73 (28.6) | 47 (18.4) | 255 (100.0) |
| **Jiangsu, China***^c^* |  |  |  |  |  |
| <=30 | 5 (12.5) | 5 (12.5) | 17 (42.5) | 13 (32.5) | 40 (100.0) |
| 31-40 | 10 (13.5) | 13 (17.6) | 30 (40.5) | 21 (28.4) | 74 (100.0) |
| 41-50 | 16 (18.6) | 23 (26.7) | 26 (30.2) | 21 (24.4) | 86 (100.0) |
| >50 | 8 (13.3) | 16 (26.7) | 24 (40.0) | 12 (20.0) | 60 (100.0) |
| Total | 39 (15.0) | 57 (21.9) | 97 (37.3) | 67 (25.8) | 260 (100.0) |
| **Freetown, Sierra Leone***^d^* | |  |  |  |  |
| <=30 | 17 (7.0) | 94 (38.5) | 99 (40.6) | 34 (13.9) | 244 (100.0) |
| 31-40 | 15 (10.3) | 72 (49.3) | 57 (39.0) | 2 (1.4) | 146 (100.0) |
| 41-50 | 14 (13.0) | 62 (57.4) | 30 (27.8) | 2 (1.9) | 108 (100.0) |
| Total | 46 (9.2) | 228 (45.8) | 186 (37.3) | 38 (7.6) | 498 (100.0) |
| **Overall** |  |  |  |  |  |
| <=30 | 41 (12.3) | 106 (31.9) | 126 (38.0) | 59 (17.8) | 332 (100.0) |
| 31-40 | 48 (16.6) | 102 (35.3) | 99 (34.3) | 40 (13.8) | 289 (100.0) |
| 41-50 | 46 (17.9) | 101 (39.3) | 78 (30.4) | 32 (12.5) | 257 (100.0) |
| >50 | 19 (14.1) | 42 (31.1) | 53 (39.3) | 21 (15.6) | 135 (100.0) |
| Total | 154 (15.2) | 351 (34.6) | 356 (35.1) | 152 (15.0) | 1013 (100.0) |

*^a^* The absolute number and the percentage of HAdV-5 nAbs in each subgroup.

*^b^* The ages of serum donors from Beijing, China, ranged from 18 to 65 years old.

*^c^* The ages of serum donors from Jiangsu, China, ranged from 18 to 60 years old.

*^d^* The ages of serum donors from Freetown, Sierra Leone, ranged from 18 to 50 years old.
